# Symptoms Associated With Personal Protective Equipment Among Frontline Health Care Professionals During the COVID-19 Pandemic

**DOI:** 10.1017/dmp.2020.455

**Published:** 2020-11-19

**Authors:** Ahmet Çağlar, İlker Kaçer, Muhammet Hacımustafaoğlu, Berkant Öztürk, Kemal Öztürk

**Affiliations:** Department of Emergency Medicine, Aksaray University Training and Research Hospital, Aksaray, Turkey

**Keywords:** COVID-19, dermatitis, headache, personal protective equipment, working performance

## Abstract

**Objective:**

Personal protective equipment (PPE) use is frequently construed as inconvenient and disturbing by health care professionals (HCPs). We hypothesized that new-onset symptoms among HCPs may be associated with extended use of PPE and aimed to investigate risk factors related with new-onset symptoms. In addition, the effects of new-onset symptoms on working performance were evaluated.

**Methods:**

In this cross-sectional study, 315 participants filled out a questionnaire that contains 4 main parts: (1) demographics, (2) new-onset symptoms with PPE use, (3) PPE usage hours, and (4) personal opinion about the effect of sensed symptoms on working performance.

**Results:**

The mean age was 31.58 ± 4.6 years, and 50.5% (n = 159) were female. New-onset symptom rate was 66% (n = 208). The most common new-onset symptom was headache (n = 115, 36.5%) followed by breathing difficulty-palpitation (n = 79, 25.1%), and dermatitis (n = 64, 20.3%). Extended use of PPE, smoking, and overweight were independently associated with developing new-onset symptoms. A clear majority of symptomatic participants pointed out the impact on working performance (193/208, 92.7%).

**Conclusion:**

Hospitals should take the necessary precautions (eg, shorter shifts and more frequent breaks) to prevent symptoms associated with PPE and ensure that HCPs comply with these precautions.

## Introduction

The 2019 coronavirus disease (COVID-19) outbreak is still affecting people worldwide. Approximately 35 million people have been infected and over 1 million people have died around the world.^1^ Person-to-person transmission mainly occurs through direct contact and indirectly through contact with contaminated surfaces.^2,3^ Potential airborne transmission depends on the patient’s clinical situation and the intervention performed on the patient.^4^ The aerosolized virus can survive up to 3 hours.^2^ Medical interventions such as non-invasive ventilation and tracheal intubation may create a localized aerosol that can allow airborne transmission to health care professionals (HCPs).^4,5^

The World Health Organization and Centers for Disease Control and Prevention published various protocols for HCPs at high risk due to interaction with COVID-19 patients.^6,7^ The World Health Organization defined personal protective equipment (PPE) as a gown, non-sterile gloves, goggles, and respirator mask.^6^ PPE use is frequently construed as inconvenient and disturbing by HCPs.^8,9^

During the pandemic, we observed some symptoms such as headache, palpitation, breathing difficulty, and dermatitis among HCPs. We hypothesized that these complaints may be associated with extended use of PPE and aimed to investigate risk factors related with new-onset symptoms. In addition, effects of new-onset symptoms on working performance were evaluated.

## Materials and Methods

This cross-sectional study was carried out between August 1 and September 1, 2020, in a tertiary pandemic hospital. During the study process, there were 212 hospitalized patients because of COVID-19 pneumonia in our hospital, and the average temperature was 22.4 °C during the study period in our region. The study was conducted in compliance with the Declaration of Helsinki and approved by Aksaray University School of Medicine, Aksaray Education and Research Hospital Scientific Research Evaluation Committee with decision no.2020/06-74.

### Study Design

There is a total of 1278 HCPs working in our hospital. HCPs who have no chronic disease are working in the isolated units for COVID-19 patients. Eligible HCPs totaling 726, using PPE and interacting with COVID-19 patients, were invited to participate in the study; 315 of them agreed to participate and signed a written consent form. All participants were reminded that they could leave the study at any time and for any reason; they were not paid any compensation.

All participants filled out a questionnaire written in Turkish and containing 4 main parts: (1) demographics (age, gender, height, weight, occupation, working unit, smoking habit); (2) new-onset symptoms with PPE use (headache, palpitation, breathing difficulty, and dermatitis) and relevant medication requirements; (3) number of days of using PPE in 1 month period and daily average of hours of PPE use; and (4) personal opinion about the effect of sensed symptoms on working performance (none – mild – moderate – severe). (See the supplementary file for the English version of the questionnaire.)

In our hospital, disposable masks (1200 N95/FFP2 NR, ERA, İstanbul, Turkey), goggles (Pulsafe LG20 Goggle, Bacou-Dalloz Company, Paris, France), isolation gowns (Safetouch TP63 5/6 classic disposable protective coverall, Safetouch Ltd, Istanbul, Turkey), and non-sterile gloves are being used routinely while caring for patients confirmed with COVID-19.

### Data Analysis

Data were analyzed using SPSS, version 22.0 (IBM Corp, Armonk, NY). Visual (histogram and probability graphs) and analytical methods (Kolmogorov-Smirnov test) were used to determine the distribution normality. Descriptive statistics were expressed as mean ± SD for normally distributed variables and as median and interquartile range for non-normally distributed variables. Categorical data were expressed as n (%). For intergroup comparisons, the Student’s t test was used to compare normally distributed data, the Mann–Whitney U test to compare non-normally distributed data, and Pearson’s chi-square or Fisher’s exact test to compare categorical variables.

We calculated body-mass index (BMI) using the kg/m^2^ formula. Participants were divided into 2 groups: overweight (BMI ≥ 25 kg/m^2^) and non-overweight (BMI < 25 kg/m^2^). Breathing difficulty and palpitation were invariably observed together, so these 2 symptoms accumulated in 1 group. A multivariate logistic regression model was constructed to determine the factors predicting new-onset symptoms among HCPs. In addition to age and gender, variables with a value of P < 0.2 in intergroup comparisons were included in the multivariate logistic regression model. All analyses were 2-tailed. A value of P < 0.05 was considered statistically significant for all analyses.

## Results

Participants totaling 50.5% (n = 159) were female and 49.5% (n = 156) were male. The mean age was 31.58 ± 4.6 years. Nurses contributed the most (n = 137, 43.5%), followed by doctors (n = 99, 31.4%) and paramedical personnel (n = 79, 25.1%). The new-onset symptom rate was 66% (n = 208). The most common new-onset symptom was headache (n = 115, 36.5%), followed by breathing difficulty-palpitation (n = 79, 25.1%) and dermatitis (n = 64, 20.3%); 69.7% (n = 145) of the participants with new-onset symptoms required medication for symptoms. A clear majority of symptomatic participants pointed out the impact of symptoms on their working performance (193/208, 92.7%). Participants’ demographic data are summarized in Table 1.

**Table 1.** Baseline characteristics of study participants (n = 315)

Female gender, n (%)	159 (50.5)
Age, years, mean ± SD	31.58 ± 4.6
Body mass index, kg/m^2^, mean ± SD	24.74 ± 3.8
Occupation, n (%)	
Doctors	99 (31.4)
Nurses	137 (43.5)
Paramedical personnel	79 (25.1)
Department, n (%)	
Emergency department	140 (44.4)
Pandemic wards and intensive care unit	175 (55.6)
Smokers, n (%)	84 (26.7)
Frequency of PPE use per month, days, mean ± SD	17.82 ± 1.8
Duration of PPE use per day, hours, mean ± SD	5.54 ± 1.2
New-onset symptoms, n (%)	208 (66)
Headache	115 (36.5)
Breathing difficulty/palpitation	79 (25.1)
Dermatitis	64 (20.3)
Drug use requirement for new-onset symptom, n (%)	145 (69.7)
Impact of new-onset symptom on overall work performance, n (%)	
None	15 (7.2)
Slightly	35 (16.8)
Moderate	50 (24)
Extreme	108 (51.9)

PPE = personal protective equipment.

A comparison of symptomatic and asymptomatic participants showed a statistically significant difference for BMI, smoking habit, frequency of PPE use per month, and duration of PPE use per day (Table 2).

**Table 2.** Comparison of participants with and without new-onset symptom

	With New-Onset Symptom (n = 208)	Without New-Onset Symptom (n = 107)	*P* Value
Female gender, n (%)	102 (64.2)	57 (35.8)	0.477
Age, years, mean ± SD	31.73 ± 4.6	31.29 ± 4.6	0.499
Body mass index, n (%)			**0.018**
< 25 kg/m^2^	97 (59.9)	65 (40.1)	
≥ 25 kg/m^2^	111 (72.5)	42 (27.5)	
Occupation, n (%)			0.831
Doctors	63 (63.6)	36 (36.4)	
Nurses	92 (67.2)	45 (32.8)	
Paramedical personnel	53 (67.1)	26 (32.9)	
Department, n (%)			0.07
Emergency department	100 (71.4)	40 (28.6)	
Pandemic wards and ICU	108 (61.7)	67 (38.3)	
Smokers, n (%)	65 (77.4)	19 (22.6)	**0.01**
Frequency of PPE use per month, days, mean ± SD	18.22 ± 1.7	17.04 ± 1.8	**0.000**
Duration of PPE use per day, hours, mean ± SD	5.73 ± 1.3	5.17 ± 1.1	**0.000**

ICU = intensive care unit; PPE = personal protective equipment.

A multivariate logistic regression analysis revealed that frequency of PPE use per month, as well as the duration of PPE use per day, was independently associated with developing new-onset symptoms. In addition to PPE usage patterns, smoking (OR = 1.93, 95% CI: 1.04–3.59, P = 0.037) and BMI (OR = 1.79, 95% CI: 1.06–3.03, P = 0.029) were associated with developing new-onset symptoms (Table 3).

**Table 3.** Multivariate logistic regression analysis of independent factors and PPE usage patterns associated with new-onset symptom

	Wald	Odds Ratio (95% Confidence Interval)	*P* Value
Age	3.041	1.05 (0.99-1.11)	0.081
Female gender	0.1	1.08 (0.64-1.84)	0.752
BMI ≥ 25 kg/m^2^	**4.746**	**1.79 (1.06-3.03)**	**0.029**
Emergency department	0.028	0.95 (0.55-1.64)	0.868
Smoking	**4.337**	**1.93 (1.04-3.59)**	**0.037**
Frequency of PPE use per month	**22.078**	**1.41 (1.22-1.64)**	**0.000**
Duration of PPE use per day	**8.312**	**1.38 (1.11-1.73)**	**0.004**

BMI = body mass index; PPE = personal protective equipment.

## Discussion

In this study, we investigated PPE-associated symptoms among frontline HCPs in Turkey during the COVID-19 pandemic. Participants totaling 66% developed PPE-associated symptoms. The extended use of PPE, smoking, and a higher BMI had a greater likelihood of developing such symptoms.

The necessity of PPE to prevent transmission to HCPs has been experienced during the severe acute respiratory syndrome (SARS) epidemic (2003), Ebola epidemic (2014), and, finally, the current COVID-19 outbreak.^4,10,11^ The PPEs used during these outbreaks and their effects on HCPs were different. During the Ebola epidemic in West Africa, where the climate is known for high ambient temperatures throughout the year, PPE effects were generally related to high temperature, and it was recommended that the duration of PPE use should not exceed 40 minutes.^12^ Heat–stress-related heat stroke, dehydration, cognitive impairment, and postural instability were the main effects of extended PPE use.^12,13^ In our study, headache, breathing difficulty/palpitation, and dermatitis were the main effects of extended PPE use for HCPs. The average temperature in our region is lower than in West Africa, and there was no symptom related to heat stress.

Lim et al. conducted a study during the SARS pandemic and reported that headache was developed in 37.3% of HCPs who were wearing N95 masks, and the duration of N95 mask usage was an important risk factor for headache development.^14^ Ong et al. confirmed this knowledge with their study during the COVID-19 pandemic. They reported that 128 of 158 (81%) HCPs developed bilateral headache relevant with PPE and that using a mask and goggles for more than 4 hours is a risk factor for headache.^15^ In a study conducted with dental professionals, Farronato et al. reported that PPE use causes headaches and breathing difficulties.^16^ They also noted that dental professionals usually take off their masks between patients, which is why time spent in PPE is not associated with symptoms.^16^ Data obtained in our study revealed that headache was the most common (36.5%) symptom associated with PPE use, and every hour spent using PPE increases new-onset symptom development 1.38 times. Nearly 70% of participants with new symptoms associated with PPE needed medication in our study. In addition, a large proportion of our participants (92.8%) experienced at least a slight decreased work performance. These rates seem higher compared to the literatüre.^15,16^ This difference may be related to the content of the PPE used and the working conditions. New-onset symptom frequency, severity, medication use, and work performance may worsen if COVID-19 cases continue to rise. Working conditions (eg, shorter shifts) should be regulated to avoid these adverse impacts of PPE use.

Farronato et al. reported that 63.5% of patients in their study population had breathing difficulties.^16^ It was a surprisingly high rate and was contrary with the literature. They mentioned that the high breathing difficulty rates were related to masks without valves.^16^ Chughtai et al. reported that the breathing difficulty rate was 12.2% in a study conducted with 148 HCPs who were using a surgical mask.^17^ Rebmann et al. analyzed the effect of N95 mask on participants and reported that some participants (21%) took off their masks after a few minutes due to breathing difficulties.^18^ In our study, all participants were using masks without valves and their breathing difficulty/palpitation ratio was 25.1% (n = 79). These 2 symptoms could be the result of CO_2_ retention caused by masks without valves. Laferty et al. investigated the oxygen and carbon dioxide levels of participants wearing N95 masks in their study, and the results showed an increase in CO_2_ levels and a decrease in SpO_2_ levels due to the significant breathing resistance caused by N95 masks.^19^ During the pandemic, increasing stress and anxiety levels were noticed among HCPs.^20,21^ Increased levels of stress and anxiety may contribute to breathing difficulty and palpitation.

Hand dermatitis due to gloves and PPE is frequently diagnosed among HCPs.^22,23^ During the pandemic, this side effect of PPE became even more important. Lan et al. reported that skin damage rates were 74.5% and 83.1% at hands and nasal bridge, respectively.^24^ A study conducted in the United Kingdom reported that mask-related pressure and the extended use of a mask were the causes in 66.3% of facial rashes among HCPs. Authors suggest that HCPs take a work break every 2 hours to prevent dermatitis.^25^ In our study, the rate of dermatitis due to PPE was 20.3%, and this rate seems lower than the literature. This low rate of dermatitis in this study may be due to the use of a disinfectant with moisturizers before wearing PPE and after hand cleaning, in our hospital.

In the present study, in addition to the extended use of PPE, smoking and BMI were also independent risk factors of new-onset symptoms. It is well-known that smoking and obesity decrease cardiopulmonary capacity and cause dyspnea.^26,27^ As expected, the use of masks without valves, making breathing difficult, and isolation gowns covering the entire body and causing continuous dehydration could lead to more symptoms in individuals who are smokers and/or obese.

### Limitations

Our study has some limitations. First, all eligible HCPs (411 HCPs did not agree to participate) could not be included in the study. This may have an effect on the symptom rates. In addition, relatively young HCPs who have no chronic disease are working in the isolated units and caring for COVID-19 patients in our hospital. The possible effects of PPE use in HCPs who have chronic diseases and are older could not be evaluated. This is a single-center study, and PPE use may differ from other hospitals. Multi-center studies with a wider age range of HCPs are needed to reveal more clearly the effects of extended PPE use.

## Conclusion

Self-protection from COVID-19 transmission is important for HCPs. A reduced workforce due to infected HCPs means extended working hours for teammates, more viral load exposure, as well as more symptoms associated with prolonged PPE use. Hospitals should take the necessary precautions (eg, shorter shifts and more frequent breaks) to prevent symptoms associated with PPE and ensure that HCPs comply with these precautions.
